# Supplementary material for: FOXO1 Couples KGF and PI-3K/AKT Signaling to NKX2.1-Regulated Differentiation of Alveolar Epithelial Cells
Source: Cells. 2022 Mar 26;11(7):1122. doi: 10.3390/cells11071122 (PMC8997990; doi:10.3390/cells11071122)
Supplement: Supplementary file 1 [file cells-11-01122-s001.zip › cells-1627884-supplementary.pdf]

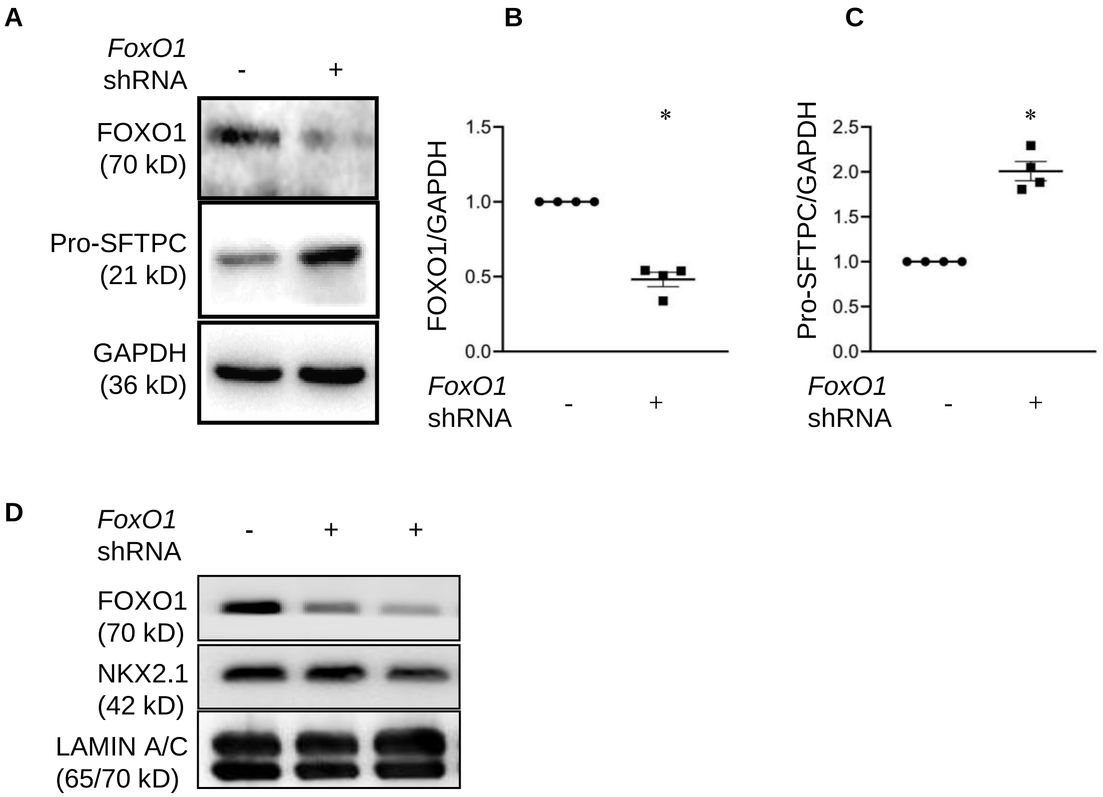

Supplementary Figure S1. Knockdown of FOXO1 in MLE-15 cells increases SFTPC but does not affect NKX2.1 expression. (A) Representative WB (A) and quantitation (B-C) show that knockdown of FOXO1 increases pro-SFTPC expression in MLE-15 cells following transduction with *FoxO1* shRNA (+) or non-silencing shRNA (-) for 4 days. GAPDH is loading control. Data are shown as normalized to non-silencing shRNA. n = 4 for each group. Unpaired two-tailed t test: * = P<0.05. (D) WB shows unchanged NKX2.1 expression in MLE-15 cells following shRNA knockdown with *FoxO1* shRNA (+) or non-silencing shRNA (-) for 4 days. n = 2.

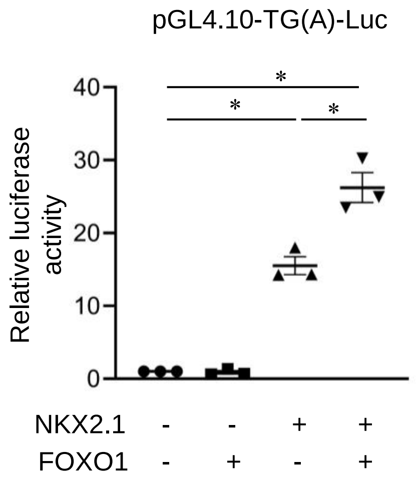

Supplementary Figure S2. FOXO1 does not inhibit NKX2.1-mediated Tg activation. Nthy-ori 3-1 cells were co-transfected with a 2.5 kb Tg reporter pGL4.10-TG(A)-Luc, NKX2.1 and FOXO1 expression constructs. Dual luciferase assays 48 h post-transduction show that FOXO1 does not repress NKX2.1 induction of the Tg reporter. Firefly luciferase activity was normalized to Renilla luciferase. Data are shown as normalized to absence of FOXO1 and NKX2 (empty vectors). n= 3 for each group. One-way ANOVA, * = P<0.05.

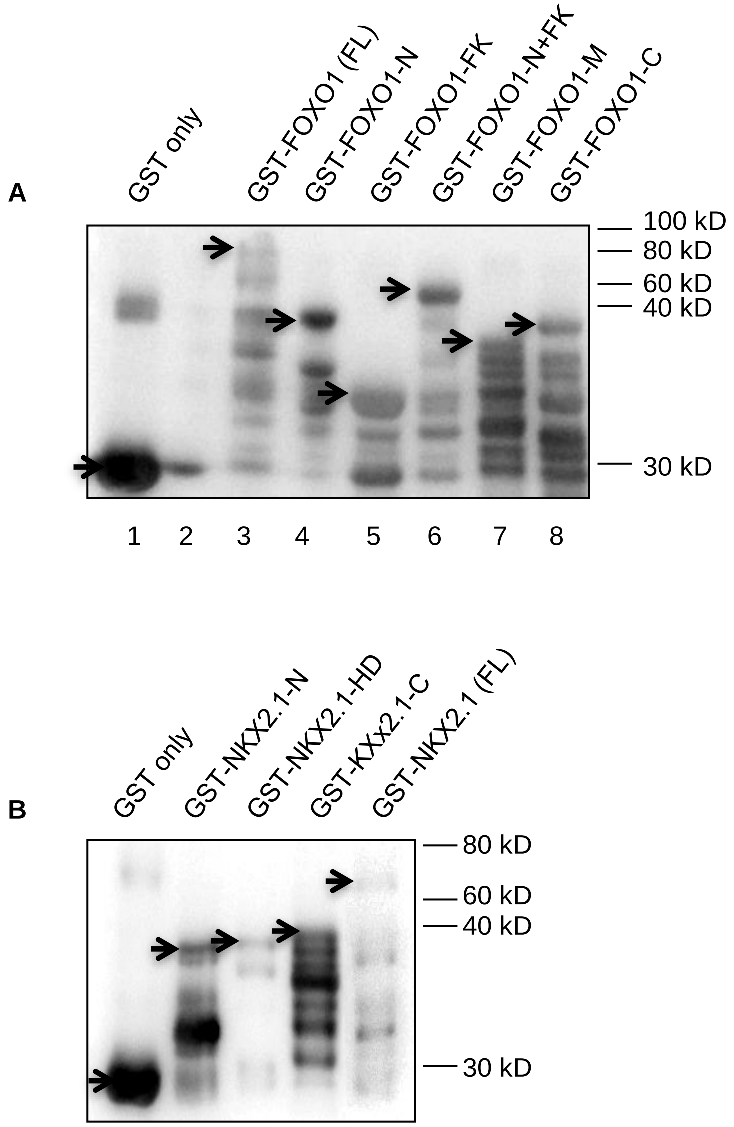

Supplementary Figure S3. **GST fusion proteins used in pull-down experiments.** (A) Western analysis using anti-GST Ab confirms expression of GST-FOXO1 fusion proteins (arrow). (B) Western analysis using anti-GST Ab confirms expression of GST-NKX2.1 fusion proteins (arrow).

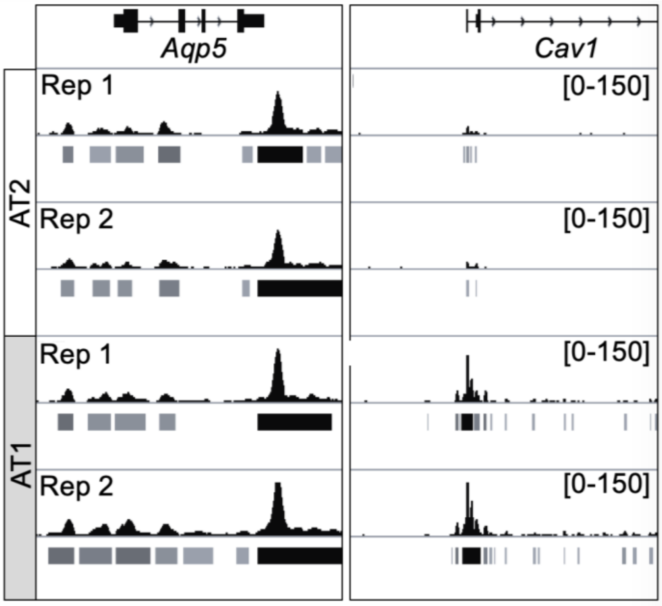

Supplementary Figure S4. Publicly available ChIPseq of NKX2-1 binding surrounding the *Aqp5* and *Cav1* loci in *Sftpc*$^{+}$ AT2 and *Wnt3A*$^{+}$ AT1 cells. Peaks were called using MACS2.0 as outlined by Little DR et al. (31). 0-150 indicates number of Chip-Seq reads overlapping at a given base.
